# Do Patients Accurately Represent Their Experiences After Hip and Knee Replacements?

**DOI:** 10.7759/cureus.12745

**Published:** 2021-01-17

**Authors:** Thomas Ashdown, Chang Park, Fahima Begum, Anna Panagiotidou, Kapil Sugand, Sherif El-Tawil

**Affiliations:** 1 Trauma and Orthopaedics, Northwick Park Hospital, London, GBR

**Keywords:** arthroplasty, patient-reported outcome measures, postoperative complications

## Abstract

Background

To investigate discrepancies, if any, between the complications that patients report on the patient-reported outcome measures (PROMs) questionnaire and what is formally recorded in their medical records.

Methodology

A retrospective analysis of PROMs-reported complications was performed at a single elective center for all patients who had an elective primary total knee or hip replacement between April 2016 and March 2017. Corresponding patient medical records were then analyzed to correlate the PROMs with any documentation of postoperative complications, which similar to the PROMs data were categorized into wound complications, urinary complications, readmission, and further operative procedures.

Results

A set of 54 complete patient records were compared to the corresponding PROMs data. The combined overall positive predictive value was 0.47 while the overall negative predictive value was 0.91. Concordance between patients and the medical records was 70.4% for wound complication, 66.7% for urinary complications, 83.3% for readmission, and 96.3% for reoperation.

Conclusion

PROMs data are becoming increasingly important in auditing and planning healthcare provision. This study highlights a significant level of discrepancy between the PROMs-reported complication rates and those documented in the medical records. There is a visible disparity between patient perception and the medical definition of postoperative complications. Further patient education and empowerment are required in preparation for arthroplasty.

## Introduction

Total hip and knee replacements are two of the most efficacious procedures in orthopedic surgery. Commonly performed, they provide excellent symptom relief and significant improvement in quality of life for those suffering from debilitating joint pain [1].

Outcomes of arthroplasty surgery can include clinical outcomes, complications, functional measurements, and patient-reported outcome measures (PROMs). PROMs came to fame as an alternative to the commonly held belief that clinical or functional metrics were the ultimate endpoint of assessing the efficacy of a service. In recent years, care has become increasingly patient-centered, which is reflected in the way operative success is measured. Rather than relying purely on clinical outcomes as judged by the surgeon, increasing emphasis is placed on the patients’ perceptions. A number of scores and assessment tools have been developed and validated to represent individual patient experiences [2,3]. These metrics are often compared preoperatively to postoperatively to measure outcome success.

In April 2009, NHS Digital started to collect PROMs in England for all NHS-funded hip and knee arthroplasty procedures [4]. Patients complete questionnaires preoperatively and at six months postprocedure. The results are collected, analyzed, and compared centrally. The PROMs questionnaires have three key domains: (i) Oxford Knee Score (OKS) or Oxford Hip Score (OHS), a joint specific tool assessing joint pain and functionality [5,6]; (ii) EuroQol-5D (EQ-5D) and visual analogue score (VAS), a generic quality of life tool [7]; (iii) and the final domain explores the patients’ perception of their surgical experience. Patients are asked to rate their overall satisfaction and if they experienced any postoperative complications. These simple yes or no questions are divided into wound complications, urinary issues, readmission to hospital, and need for secondary operative procedures or revision.

Currently, the outcomes of the PROMs data are published annually by NHS Digital and are available for all to view in the public domain. The PROMs outcomes are audited and are influential in changing practice. However, as with any audit of outcomes, the conclusions drawn are only as reliable as the quality of data that is submitted. Any discrepancy in the data can have implications in incorrectly suggesting outliers but also masking true outliers. The data also has wider-reaching implications in terms of commissioning and healthcare at a wider policy level [8,9].

The National Joint Registry (NJR) is the world’s largest arthroplasty registry and closely audits the outcomes of elective hip and knee replacements including the performance of individual implants, surgeons, and institutions. The NJR has been central in identifying issues such as the high failure rates in metal-on-metal hip replacements and continues to identify outliers in performance of implants and institutions [10]. The NJR recognizes that clinical outcomes alone are not sufficient in assessing the efficacy of an arthroplasty procedure. From January 2018 the NJR, via the NJR PROMs Working Group, began to collect and publish the PROMs data for all patients within the registry [4]. With this increased emphasis on PROMs, the accuracy and validity of PROMs data are becoming ever more important [8,11].

To the authors’ knowledge there have been no studies comparing the PROMs reported to NHS Digital to those actually experienced during the patient journey. The OKS, OHS, EQ-5D, and VAS tools have all undergone formal validation [5-7]. However, the validity in which patients answer PROMs questions relating to complications have not undergone similar scrutiny. This study aimed to investigate if there was a discrepancy between the complications that patients report on the PROMs questionnaire to those formally recorded in their medical records.

## Materials and methods

A retrospective analysis of patient records was performed at a single elective center for arthroplasty surgery. Data were reviewed for those who had an elective primary total knee or hip replacement from April 2016 to March 2017 for all arthroplasty surgeons. Those who had revision surgery or unicondylar knee replacements (as excluded from PROMs program analysis) were excluded from this study [12].

PROMs data for this period were acquired from NHS Digital databases, accessed by the trust’s clinical governance department. The PROMs data were analyzed for patient-reported complications. Complications were categorized into wound complications, urinary complications, readmission, and further operative procedures. The data collection was performed by four clinicians in the orthopedic department and stored on the trust server.

All corresponding patient medical records were then analyzed after corroborating with the NHS Digital dataset on PROMs. This included the physical medical notes, the electronic data stored for emergency department admissions, and the trust’s electronic medical records system that stored all clinic appointment notes along with community nursing visits. All patients were routinely followed up for two subsequent weeks postoperatively by a community-based team, made up of general practitioners, physiotherapists, district nurses, and occupational therapists. Notes from such community visits were accessed from the trust’s electronic medical records system. The hospital’s admission system was also checked for any readmissions.

The medical records were analyzed for patient demographics, including age, gender, and length of stay. All records were then reviewed for postoperative complications as categorized by the PROMs data into (i) wound complications, (ii) urinary complications, (iii) readmission, and (iv) further operative procedures.

Documented wound complications were subdivided as follows: stained dressing, wound hematoma, wound dehiscence, and recorded wound infection. The clinical diagnosis of infection was judged by the clinician including clinical presentation, biochemical markers, and wound swab cultures. Furthermore, it was noted if the patient required treatment with antibiotics or if any further operative intervention was necessary. Dressing staining, in the absence of pus, was not deemed to be a true clinical wound complication but rather a normal consequence of any operative intervention.

Documented urinary complications were similarly subdivided into patients who had an episode of recorded retention or infection. The clinical diagnosis of infection was also based on the clinician’s judgment taking into account urine laboratory cultures, urinary dipstick tests, biochemical markers, and clinical presentation. It was also recorded if the patient required a urinary catheter or a course of antibiotics.

For those who required readmission or further operative intervention, the nature of presentation and diagnosis was recorded along with the outcome of care.

Statistical analysis

PROMs questionnaire answers were compared to the patient’s medical records, which were regarded as the gold standard. Data was inserted into a 2 × 2 confusion matrix. The positive predictive value (PPV) and negative predictive value (NPV) were calculated signifying the likelihood of the patient correctly reporting the presence or absence of complication. The sensitivity (ability to correctly identify the presence of a true complication), specificity (ability to correctly identify the absence of a complication), and concordance (agreement between patients and medical records) were also calculated.

## Results

**Figure 1:**
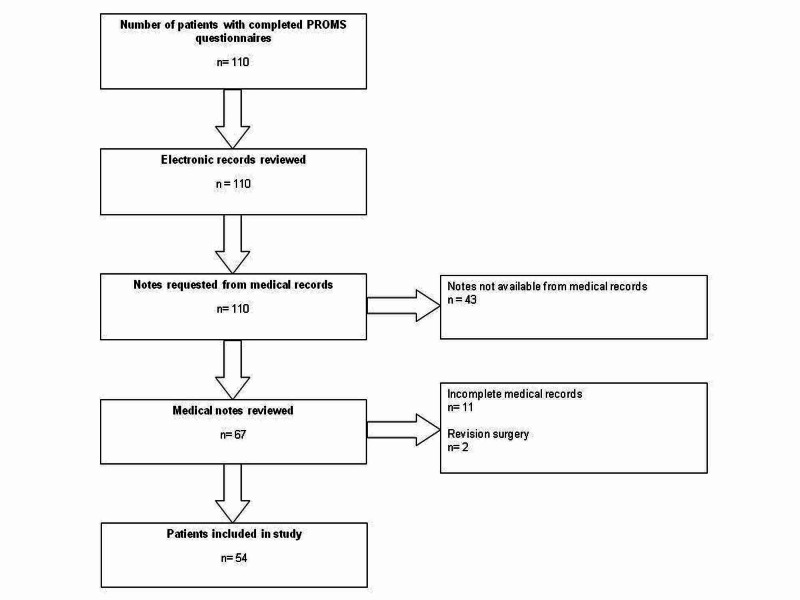
Study attrition flowchart.

Across the study period, 110 patients had completed both the preoperative and postoperative PROMs questionnaires. All 110 patient electronic records were analyzed for any documented evidence of complications. The corresponding physical medical notes were requested from medical records and 67 were made available. However, 11 notes were incomplete and excluded from the study. Two patients were identified as having had revision surgery and were excluded from the study. As a result, 54 patients were included in the study (Figure 1).

The average age of those included in the study was 68 years, with 70% (n = 38) being female and 30% (n = 16) male. A total of 81% (n = 41) of the patients had a total knee replacement and 19% (n = 10) had a total hip replacement. A total of 59 complications were reported by 47 patients on the PROMs questionnaires. There was evidence of 42 complications in the medical records, which have been detailed in Tables 1-4.

**Table 1:** Documented wound complications.

Wound complication	12 (22.2% of patients)
Superficial infection	10
Infection + dehiscence	1
Hematoma + dehiscence	1

**Table 2:** Documented urinary complications.

Urinary complication	13 (24.1% of patients)
Urinary retention	8
Urinary tract infection	3
Hematoma + Dehiscence	2

**Table 3:** Documented readmissions.

Readmission	12 (22.2% of patients)
Wound complication	6
Unrelated medical problem	3
Dislocation	1
Deep-seated infection	1
Stiffness	1

**Table 4:** Documented reoperations.

Reoperation	5 (9.3%)
Manipulation under anesthesia	2
Wound debridement	2
Revision	1

The PPV, NPV, sensitivity, and specificity of the four complication domains are detailed in Table 5. The combined overall PPV was 0.47 while the overall NPV was 0.91. Concordance between patients and the medical records was 70.4% for wound complication, 66.7% for urinary complications, 83.3% for readmission, and 96.3% for reoperation.

**Table 5:** The PPV, NPV, sensitivity, and specificity of patient-reported complications compared to the documented complications. PPV, positive predictive value; NPV, negative predictive value

	Wound Complications	Urinary Complications	Readmission	Reoperation
PPV	0.4	0.37	0.6	0.8
NPV	0.88	0.83	0.92	0.98
Sensitivity	0.67	0.54	0.75	0.8
Specificity	0.71	0.71	0.86	0.98

## Discussion

Outcome data in orthopedics has revolutionized the speciality and led the way in improving patient care and safety while simultaneously allowing surgeons and institutions to review and audit their outcomes. The NJR has been at the forefront of this, with its focus on revision rates and mortality. PROMs data collected by NHS Digital takes this a step further, looking at patient satisfaction and experience following such surgery. A best practice tariff is now payable to NHS hospitals that meet a certain standard in PROMs results as well as NJR compliance; hence, increasing the accuracy of such data is financially important as well [9].

However, with increased use and reliance on such data in audit and research, there has been increased scrutiny and work in the validity of data within orthopedics. Some questions have been raised about the completeness and quality of the data published [11]. There have been large-scale studies to assess the accuracy of data within the NJR for metal-on-metal hip arthroplasty with some aspects of code incorrectly recorded in as high as 16.6% of cases and incomplete records at rates of 39.1% [13]. A study by Howes et al. investigating the accuracy of anesthetic data in 100 patients found 52% had a discrepancy between the NJR data recorded and that found in the anesthetic charts within the medical notes [14].

This is the first study, to the authors’ knowledge, demonstrating discrepancies between complications reported in NHS PROMs data to that documented in the patient medical records. Patient reporting demonstrated a poor PPV and sensitivity indicating that patients cannot reliably report the incidence of complications. This was most evident when patients were reporting the presence of urinary or wound complications. Conversely, patients appeared to more accurately report the absence of complications with high NPV and specificity values observed. Our data is in keeping with an Australian registry study that demonstrated patients more accurately report the absence of complications but not the presence [15].

The inconsistency between the medical records and the PROMs data may be due to recall bias due to patients completing the questionnaire six months postoperatively. A study has shown that even three months following a total knee replacement, patients recall of their level of preoperative pain was poor [16]. Patients at six months postoperatively may, due to recall bias, have a differing recollection of events that may produce discrepancy to that of the contemporaneously recorded medical notes.

There may also be differences in perception as to what patients constitute a “complication” [17]. Nine patients, who had a documented staining of the dressing, reported a “wound complication” in their PROMs questionnaire. These patients may have referred to the dressing staining as a wound complication when clinicians would count this as a normal postoperative finding. Unfortunately, the national questionnaire does not define “wound complications” for patients. Previous studies have demonstrated a significant difference in the accuracy of patients reporting complications depending on the type of complication being reported [18-20]. Many studies have shown patients to be poor at self-diagnosing surgical site infections which may partly explain the low PPVs seen in category [21-23]. However, it is difficult to apply this theory of varying perceptions to the outcomes of readmission or reoperation which are clearer and more binary outcomes.

A strength of this study was the comprehensive search of patient records, both physical and electronic, from different hospital departments and professionals, to allow an accurate portrayal of complications encountered. However, despite these attempts, it is possible that some patients could have presented to another hospital or to their own general practitioners with complications that would escape our data capture. We tried to reduce this by a comprehensive search of patient records that involved community support services. Also, as part of the postoperative follow-up in outpatient clinic, patients are reviewed at regular intervals, and any postoperative complications treated outside of the trust should have been highlighted at this point.

This study was limited by the small sample size of 54 patients caused by two main factors: the limited number of complete PROMs data available (fully completed pre and postoperative questionnaires) and the difficulty in obtaining complete patient medical records. The poor PROMs response rate may be due to demographic factors. Ho et al. identified younger age and an English-speaking background as factors that improved PROMs response rate [24]. Our hospital serves a diverse population where a high proportion of people speak English as a second language. This may have impacted our response rate and the quality of data received. A study by El-daly et al. suggested most orthopedic PROMs were incomprehensible to the average UK adult [25]. A further limitation is, just as we rely on patient accuracy in reporting PROMs, we also rely on medical staff documenting complications accurately in the patient’s medical notes. However, the authors believe that despite being a small cross-section of the arthroplasty cohort, a significant issue has been highlighted that justifies further review.

## Conclusions

This study has highlighted clear discrepancy between the PROMs complication rates reported by NHS Digital and that documented on review of the medical records. As PROMs data are set to become increasingly influential in both clinical practice and strategic planning, it is imperative that the data represent the true outcome. If patient-reported complications are to continue as a domain in the PROMs questionnaire, changes should be made to ensure the data is relevant and accurate. Further patient education and empowerment may improve patient expectations of postoperative course. Although this study is only based on a single center with a limited number of patients, the findings suggest that more work is warranted in this field.
